# Rotavirus Infection and Cytopathogenesis in Human Biliary Organoids Potentially Recapitulate Biliary Atresia Development

**DOI:** 10.1128/mBio.01968-20

**Published:** 2020-08-25

**Authors:** Sunrui Chen, Pengfei Li, Yining Wang, Yuebang Yin, Petra E. de Ruiter, Monique M. A. Verstegen, Maikel P. Peppelenbosch, Luc J. W. van der Laan, Qiuwei Pan

**Affiliations:** a Department of Gastroenterology and Hepatology, Erasmus MC-University Medical Center, Rotterdam, The Netherlands; b Department of Surgery, Erasmus MC-University Medical Center, Rotterdam, The Netherlands; Corporación CorpoGen

**Keywords:** biliary atresia, rotavirus infection, human organoids

## Abstract

Biliary atresia (BA) is a neonatal liver disease characterized by progressive fibroinflammatory obliteration of both intrahepatic and extrahepatic bile ducts. The etiologies of BA remain largely unknown, but rotavirus infection has been implicated at least for a subset of patients, and this causal relation has been well demonstrated in mouse models. In this study, we aim to further consolidate this evidence in human biliary organoids. We obtained seven batches of human biliary organoids cultured from fetal liver, adult liver, and bile duct tissues. We found that these organoids are highly susceptible and support the full life cycle of rotavirus infection in three-dimensional culture. The robust infection triggers active virus-host interactions, including interferon-based host defense mechanisms and injury responses. We have observed direct cytopathogenesis in organoids upon rotavirus infection, which may partially recapitulate the development of BA. Importantly, we have demonstrated the efficacy of mycophenolic acid and interferon alpha but not ribavirin in inhibiting rotavirus in biliary organoids. Furthermore, neutralizing antibody targeting rotavirus VP7 protein effectively inhibits infection in organoids. Thus, we have substantiated the causal evidence of rotavirus inducing BA in humans and provided potential strategies to combat the disease.

## Abstract (precis)

There is substantial evidence indicating the possible involvement of rotavirus in biliary atresia (BA) development, at least in a subset of patients, but concrete proof remains lacking. In a mouse model, it has been well demonstrated that rotavirus can infect the biliary epithelium to cause biliary inflammation and obstruction, representing the pathogenesis of BA in humans. By using recently developed organoids technology, we now have demonstrated that human biliary organoids are susceptible to rotavirus infection, and this provokes active virus-host interactions and causes severe cytopathogenesis. Thus, our model recapitulates some essential aspects of BA development. Furthermore, we have demonstrated that antiviral drugs and neutralizing antibodies are capable of counteracting the infection and BA-like morphological changes, suggesting their potential for mitigating BA in patients.

## OBSERVATION

Biliary atresia (BA) is characterized by progressive fibroinflammatory obliteration of the bile ducts, resulting in chronic cholestasis and biliary cirrhosis. It is one of the leading causes for liver transplantation in infants (1, 2). Exposure to rotavirus in mice has demonstrated the infection in biliary epithelium, resulting in BA-like biliary inflammation and obstruction (3). Nevertheless, whether rotavirus is a causal agent for BA in patients remains controversial, also because of a paucity of preclinical models. Organoid technology provides an excellent way forward here. These three-dimensional (3D) cultured organoids are superior in recapitulating the architecture, composition, diversity, organization, and functionality of cell types of the tissue/organ of origin. Human organoids have been increasingly explored to advance research in disease modeling (4, 5). Although it is feasible to culture hepatocyte-like organoids from liver tissue, it remains technically challenging, with requirements for stringent experimental protocols (6). In contrast, organoids resembling the cholangiocyte phenotype are relatively easy to culture from the hepatic and extrahepatic bile duct compartments (7–9). In this study, we explored the feasibility of employing human biliary organoids cultured from fetal liver, adult liver, and bile duct for recapitulating BA development.

**FIG 1:**
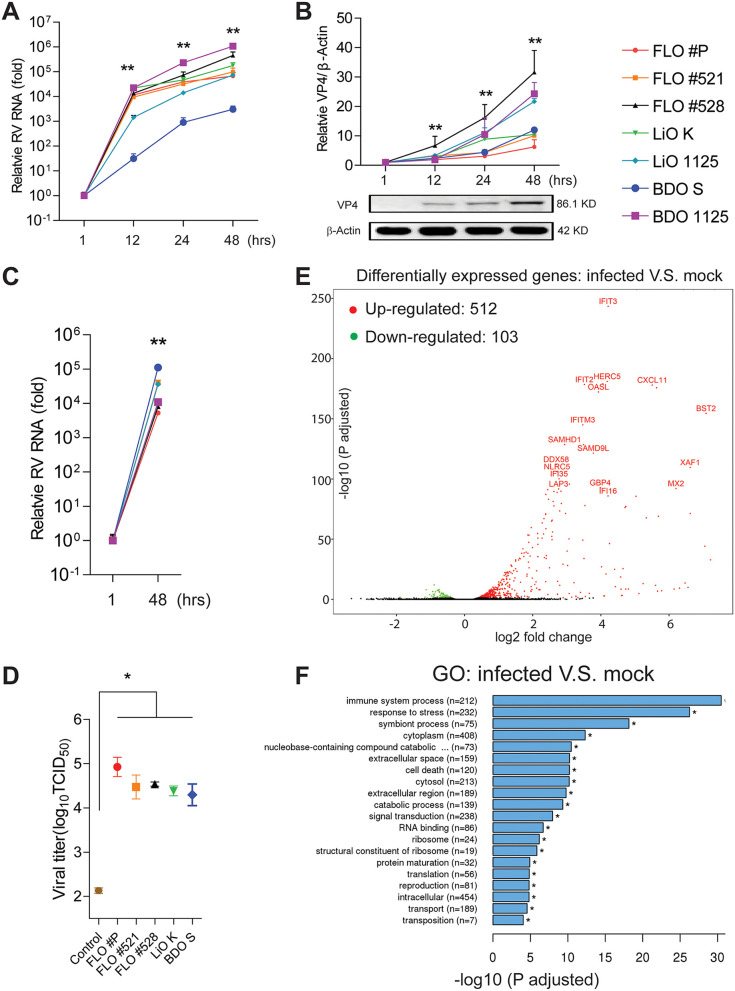
Characterizing rotavirus infection in human biliary organoids. (A) Dynamics of cellular viral RNA levels upon inoculation of SA11 rotavirus at different time points postinoculation. The level at 1 h postinoculation was set as 1. Three batches of human fetal liver organoids (FLOs), two adult liver organoids (LiOs), and two bile duct organoids (BDOs) were tested. Symbols and colors as for panel B. (B) Expression of rotavirus VP4 protein in the organoids determined by Western blotting. (C) Inoculation of human intestinal Caco2 cell line with supernatant from rotavirus-infected organoids for 48 h. Relative cellular viral RNA levels were quantified. (D) TCID_50_ of five batches of rotavirus-infected organoids at 48 h postinoculation compared with the basal level at incubation. Organoids after inoculation were thoroughly washed to remove free viruses and subjected to repeated freezing and thawing to harvest the attached and entered rotaviruses. The total amounts of rotaviruses in organoids incubated for 48 h were harvested by repeated freezing and thawing of the entire well. (E) Volcano plots of differentially expressed genes in rotavirus-infected (for 48 h) compared to uninfected fetal liver organoids. (F) Gene ontology (GO) enrichment analysis of differentially expressed genes. All data are presented as means ± standard errors of the means (SEMs). For each organoid batch, experiments were repeated 3 to 6 times. *, *P* < 0.05; **, *P* < 0.01 by Mann-Whitney test.

The canonical compartment for rotavirus infection is the small intestinal enterocyte. We previously showed that human intestinal organoids (HIOs) sustain rotavirus infection (4), and we confirmed these results (see Fig. S1A to C in the supplemental material). BA is a disorder that typically first manifests itself during mid-gestation, and murine experimentation has demonstrated rotavirus-induced BA development. Hence, we first tested if biliary fetal liver organoids (FLOs) support rotavirus infection. Inoculation of FLOs with rotavirus resulted in an increase of cellular viral RNA by a factor of 10^3^ to 10^5^ at 24 h and 10^4^- to 10^6^-fold at 48 h postinoculation (Fig. 1A) with a concomitant increase in levels of rotavirus VP4 protein (Fig. 1B). Thus, the human fetal biliary epithelium is highly permissive for rotavirus infection comparable to the level in intestinal epithelium (Fig. S1A). In apparent agreement, supernatant harvested from infected FLOs effectively infected and replicated in a Caco2 intestinal epithelial model as shown by reverse transcription-quantitative PCR (qRT-PCR) of viral RNA (Fig. 1C). Next, we performed a 50% tissue culture infective dose (TCID_50_) assay to compare the levels of infectious viral particles between the baseline of inoculation and five batches of organoids at 48 h postinoculation. We harvested rotavirus from the organoids through repeated freezing and thawing and demonstrated 10^2^- to 10^3^-fold increase of infectious virus titers (Fig. 1D). This was further confirmed by cytopathic effects in Caco2 cells at 48 h postinoculation with rotavirus harvested from these five batches of organoids and the control (see Fig. S2 in the supplemental material). Collectively, these results convincingly showed effective replication and production of infectious viral particles by infected fetal biliary organoids. Similar results were obtained in biliary organoids derived from adult human liver and bile duct (Fig. 1A to C). Thus, the human biliary epithelium is highly susceptible to rotavirus infection and supports its full life cycle.

10.1128/mBio.01968-20.1 FIG S1 Characterizing rotavirus infection in human intestinal organoids (HIOs). (A) Rotavirus RNA quantified by qRT-PCR postinoculation. (B) Optical microscopy images of infected and uninfected organoids (top). Fluorescence staining of dead cells (PI; red), live cells (calcein; green), and nuclei (Hoechst; blue) (middle). Confocal immunostaining of rotavirus structural protein VP6 (red), Epcam (green), and nuclei (blue) (bottom). (C) Quantitative analysis of the percentage of deteriorated organoids with or without rotavirus infection at indicated time points and calculated based on LIVE/DEAD cell staining (F, middle). (D) The effects of the broad-spectrum antiviral drugs on rotavirus in intestinal organoids. Rib, ribavirin; MPA, mycophenolic acid; IFN-α, interferon alpha. All data are presented as means ± SEMs. Experiments were repeated 3 to 6 times. *, *P* < 0.05; **, *P* < 0.01 by Mann-Whitney test. Download FIG S1, PDF file, 0.4 MB. Copyright © 2020 Chen et al. 2020 Chen et al. This content is distributed under the terms of the Creative Commons Attribution 4.0 International license.

10.1128/mBio.01968-20.2 FIG S2 Optical microscopy images of Caco2 cells infected with 10^4^-fold-diluted rotavirus (RV) stocks harvested from five batches of organoids infected with rotavirus at 48 h postinfection, with the control at baseline inoculation (see Fig. 1D for details). In the mock group, cells were all hyaline and polygonous, while cells in the RV-infected group, except the controls, were spindle shaped or crimpled, with large amounts of exfoliated cells, indicating cytopathogenesis. Download FIG S2, PDF file, 0.6 MB. Copyright © 2020 Chen et al. 2020 Chen et al. This content is distributed under the terms of the Creative Commons Attribution 4.0 International license.

To better understand the consequences of rotavirus infection in biliary epithelium, we performed a genome-wide transcriptomic analysis of FLOs upon infection. Volcano plots of the results showed significant downregulation of 103 and upregulation of 512 genes in response to rotavirus compared to that in uninfected organoids (Fig. 1E). Most of the highly upregulated genes, including *IFIT2*, *IFITM3*, *OASL*, *DDX58*, *MX2*, *IFI35*, *HERC5*, and *BST2*, are interferon-stimulated genes (ISGs). Other genes, such as *CXCL11* and *NLRC5* are related to the inflammatory response. Gene ontology (GO) enrichment analysis of these differentially expressed genes confirmed the essential involvement of the “immune system process” (Fig. 1F). Interestingly, “response to stress,” “cell death,” and “extracellular space” were also identified as the top regulated processes, with obvious relations to the development and pathogenesis of BA (Fig. 1E).

**FIG 2:**
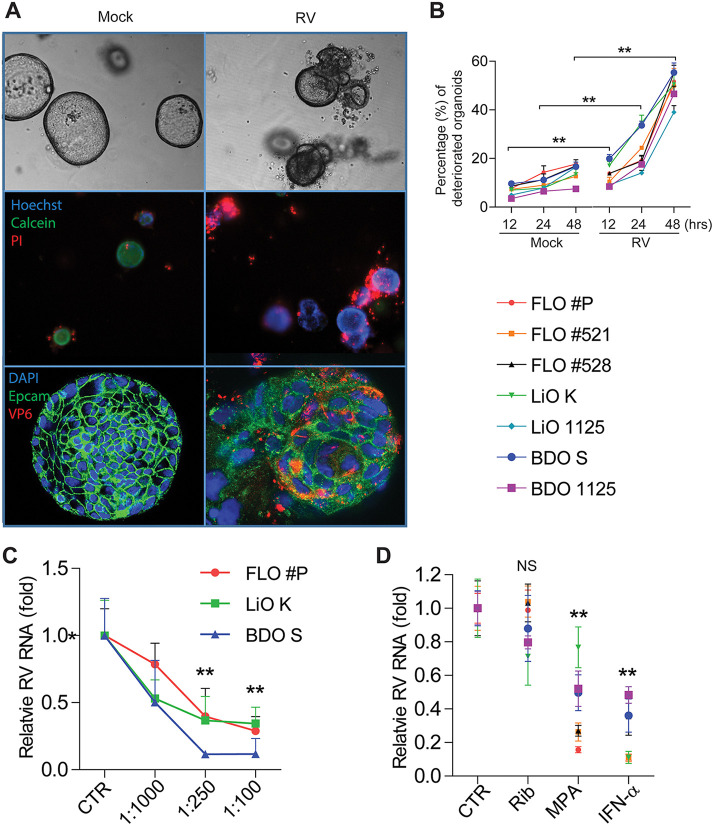
Cytopathogenesis of rotavirus-infected human biliary organoids and efficacy of antiviral treatment/neutralizing antibody. (A) Organoids from 50 μm to 150 μm in diameter were selected to capture images. Optical microscopy images of infected and uninfected organoids (top). Fluorescence staining of dead cells (PI; red), live cells (calcein; green), and nuclei (Hoechst; blue) (middle). Confocal immunostaining of rotavirus structural protein VP6 (red), epithelial cell adhesion molecule (Epcam; green), and nuclei (blue) (bottom). These are representative images of one FLO batch from the tested seven biliary organoid batches. (B) Quantitative analysis of the percentage of deteriorated organoids with or without rotavirus infection at indicated time points and calculated based on LIVE/DEAD cell staining (A, middle). (C) The inhibitory activities of neutralizing monoclonal antibody HS-1 against rotavirus infection in three representative batches of biliary organoids. (D) The effects of the broad-spectrum antiviral drugs on rotavirus in biliary organoids. Rib, ribavirin; MPA, mycophenolic acid; IFN-α, interferon alpha. All data are presented as means ± SEMs. For each organoid batch, experiments were repeated 3 to 6 times. NS, not significant; **, *P* < 0.01 by Mann-Whitney test.

This is in line with the observations that naive organoids grow and become hyaline in a spheroidal shape, whereas rotavirus-infected organoids are opaque, shriveled, and disorganized (Fig. 2A, top). Propidium iodide (PI) staining marked the wide spread of dead cells in infected organoids (Fig. 2A, middle). Confocal analysis after immunostaining of viral VP6 protein further visualized the disruption of infected organoid cells (Fig. 2A, bottom). Quantitative analysis demonstrated significant increases of the percentages of deteriorated biliary organoids at 12, 24, and 48 h postinfection of rotavirus (Fig. 2B). Thus, rotavirus infection causes severe cytopathogenesis in human biliary organoids.

Next, we evaluated a monoclonal neutralizing antibody targeting rotavirus VP7 protein (10) by using three representative batches of biliary organoids. It effectively inhibited rotavirus infection in a dose-dependent manner (Fig. 2C). Finally, the effects of the known broad-spectrum antiviral drugs were tested in all batches of organoids. Similarly to that in HIOs (see Fig. S1D in the supplemental material), mycophenolic acid (MPA) and interferon alpha (IFN-α) potently inhibited rotavirus in all batches of biliary organoids (Fig. 2D). Surprisingly, ribavirin was effective in intestinal (Fig. S1D) but not in biliary (Fig. 2D) organoids. Therefore, antiviral drugs and neutralizing antibodies are potential therapeutics to combat rotavirus infection in the human biliary epithelium compartment.

### Discussion and conclusions.

Although the etiologies and pathogenesis of BA remain largely unknown, multiple pathogenic mechanisms are likely involved, including genetic mutations (11), exposure to environmental toxins (12), dysregulation of the immune system, and, most intriguingly, viral factors in a particular rotavirus (3, 13–18). Previous studies have attempted to detect rotavirus in liver or biliary tissues and the antibody in sera from BA patients, but results are inconclusive (19). Since the wide implementation of vaccines that have substantially counteracted rotavirus-mediated diarrheal disease, a more direct investigation on the causality of rotavirus infection for BA has become possible. A survey of the national registry system in Taiwan found a decreased incidence of BA from 2004 to 2009, mirroring the increased uptake of rotavirus vaccination (20). A nationwide population-based study in Korea has shown that rotavirus infection in neonates is a risk factor for BA, although vaccination did not impact disease incidence (21).

Unfortunately, detection of rotavirus in tissue is often not feasible, as advanced disease is usually diagnosed in children 4 to 6 weeks old and the virus likely has been cleared by that time. Here we show, using organoid technology, that the human biliary epithelium supports the full life cycle of rotavirus infection and results in cellular and morphological changes consistent with BA development, even in the absence of immune cell components in our model. Furthermore, we identify therapeutic strategies potentially useful for combating rotavirus infection in the biliary epithelium.

Interestingly, a study in mice has demonstrated that maternal vaccination can prevent rotavirus-induced BA in newborn pups (22). This is in line with our findings that neutralizing antibodies inhibit rotavirus infection in organoids. Thus, we have substantiated the causal evidence of rotavirus inducing BA in humans and provided potential strategies to combat the disease.

### Materials and methods.

Human fetal liver organoids (FLOs; *n *= 3 batches) were initiated from 17-week-old human fetal livers collected at abortion, from adult liver (LiOs; *n *= 2 batches), and from adult bile duct (BDOs; *n *= 2 batches). Human intestinal organoids (HIOs; *n *= 1 batch) were cultured to serve as a standard model for rotavirus infection. Detailed methods are described in Text S1.

10.1128/mBio.01968-20.3 TEXT S1 Supplemental materials and methods. Download Text S1, PDF file, 0.1 MB. Copyright © 2020 Chen et al. 2020 Chen et al. This content is distributed under the terms of the Creative Commons Attribution 4.0 International license.

### Data availability.

The complete dataset was deposited at Data Archiving and Networked Services (DANS) (https://easy.dans.knaw.nl/ui/datasets/id/easy-dataset:179259; https://doi.org/10.17026/dans-27b-j8k9) (see Text S1 for details).
