# Supplementary figures and images for: Rotavirus Infection and Cytopathogenesis in Human Biliary Organoids Potentially Recapitulate Biliary Atresia Development
Source: mBio. 2020 Aug 25;11(4):e01968-20. doi: 10.1128/mBio.01968-20 (PMC7448284; doi:10.1128/mBio.01968-20)

**A**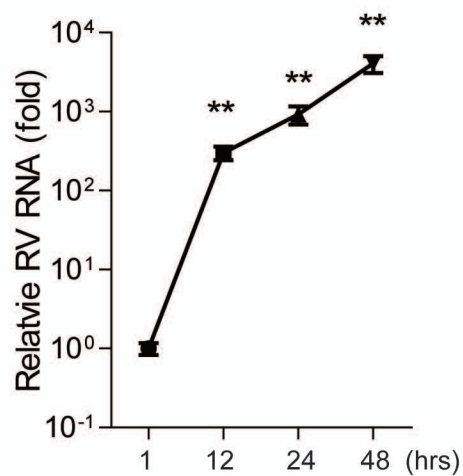**C**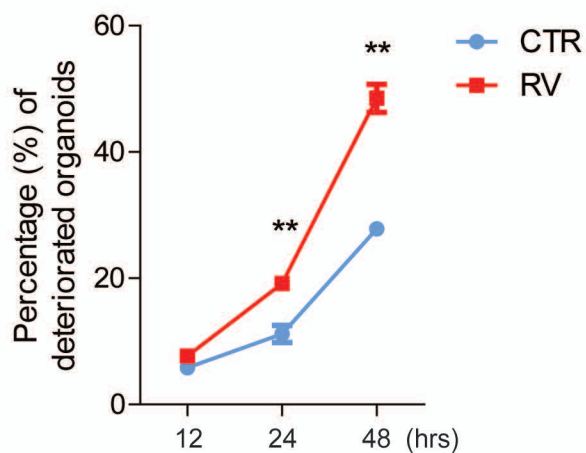**D**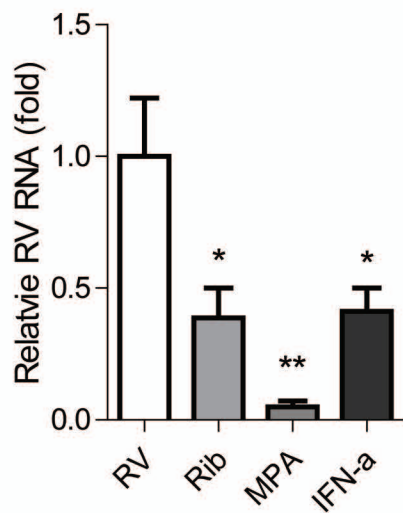**B**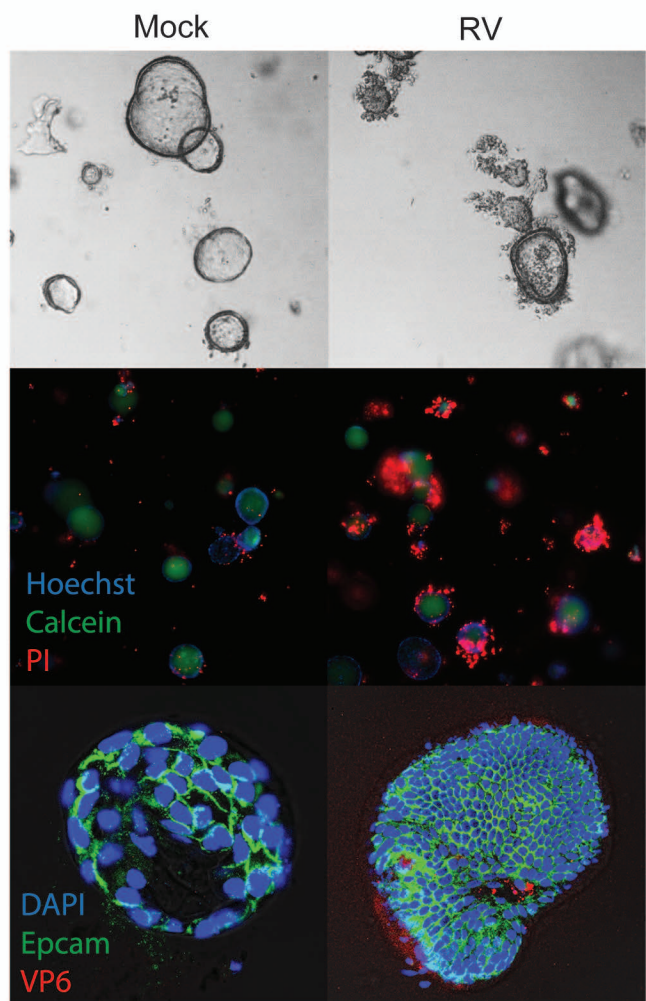

Supplement: FIG S1 [file mBio.01968-20-sf001.pdf]

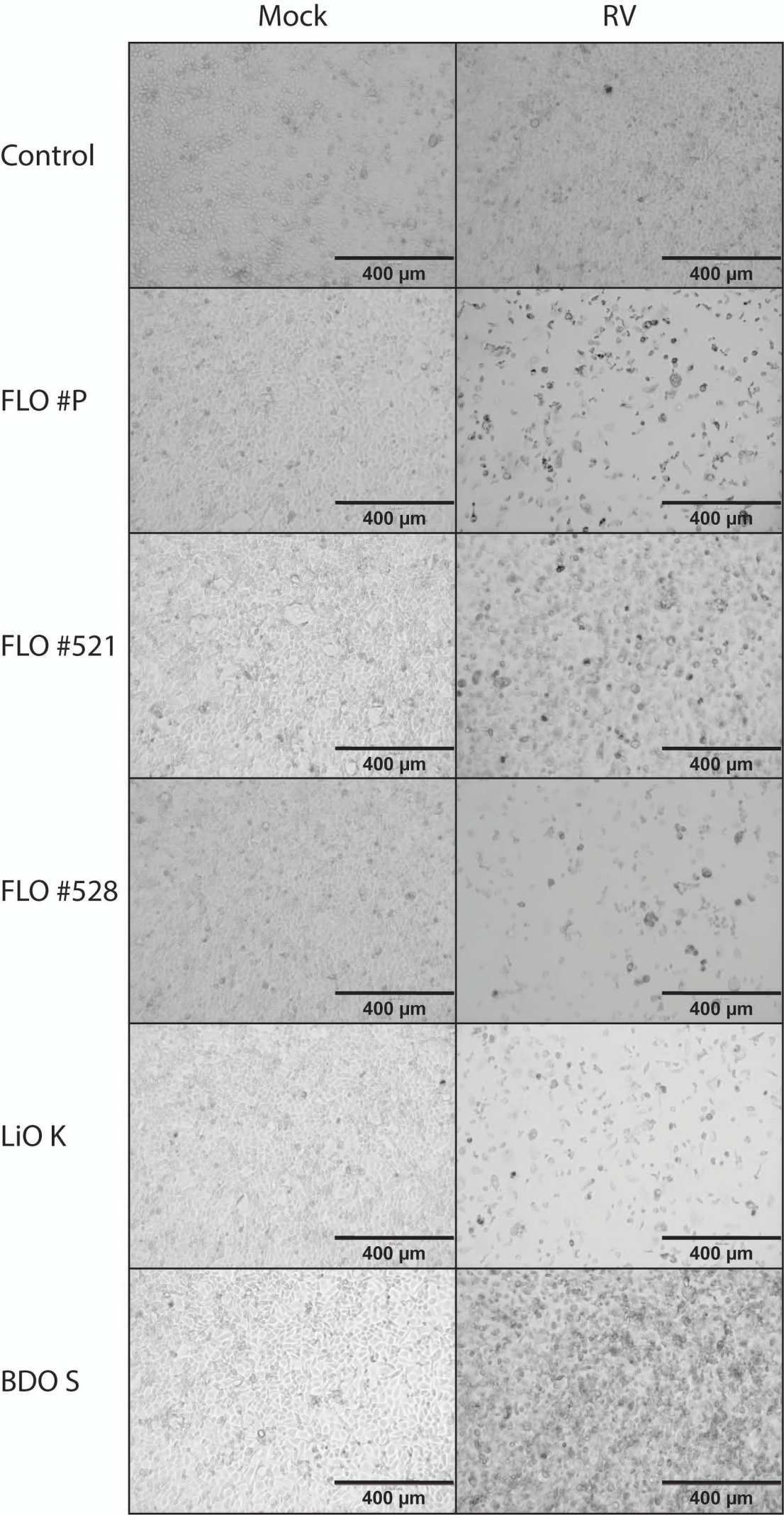

Supplement: FIG S2 [file mBio.01968-20-sf002.pdf]
