# Supplementary material for: Rotavirus Infection and Cytopathogenesis in Human Biliary Organoids Potentially Recapitulate Biliary Atresia Development
Source: mBio. 2020 Aug 25;11(4):e01968-20. doi: 10.1128/mBio.01968-20 (PMC7448284; doi:10.1128/mBio.01968-20)
Supplement: TEXT S1 [file mBio.01968-20-s0001.pdf]

Rotavirus infection and cytopathogenesis in human biliary organoids potentially recapitulate biliary atresia development

Sunrui Chen[1], Pengfei Li[1], Yining Wang[1], Yuebang Yin[1], Petra E. de Ruiter[2], Monique M. A. Verstegen[2], Maikel P. Peppelenbosch[1], Luc J. W. van der Laan[2], Qiuwei Pan[1]*

[1]Department of Gastroenterology and Hepatology, Erasmus MC-University Medical Center, Rotterdam, The Netherlands.

[2] Department of Surgery, Erasmus MC-University Medical Center, Rotterdam, The Netherlands.

Text S1 – Supplemental Materials and Methods

Reagents

Propidium iodide (PI) (Method Detection Limit [MDL] no. MFCD00011921), calcein-AM (MDL no. MFCD05861516), ribavirin (MDL no. MFCD00058564) and mycophenolic acid (MPA) (MDL no. MFCD00036814) were purchased from Sigma. All the reagents above were dissolved in dimethyl sulfoxide (DMSO). Hoechst 33342 (Catalog number: H3570) and Type I human recombinant IFN alpha 2a (IFN-α) were purchased from Thermo Fisher, and IFN-α was dissolved in culture medium.

Viruses

Simian rotavirus SA11, a widely used laboratory strain(1), was gifted by Karen Knipping from Nutricia Research Utrecht, The Netherland. Rotavirus SA11 was prepared as previously described(2).

Cell lines and human organoids

Human colon cancer cell line Caco2 was cultured as previous study(3). Cells were analyzed by genotyping and confirmed to be mycoplasma negative.

Human primary small intestinal organoids (HIOs) were cultured as described previously(4). three batches of human fetal liver organoids (FLO P, FLO 521 and FLO 528), two adult bile duct organoids (BDO S, DD 1125) and two adult liver organoids (LiO K, DL 1125) were cultured as previously described(5). The use of human organoids was approved by the Medisch Ethische Toetsings Commissie Erasmus MC (Medical Ethical Committee of the Erasmus medical center).

Virus inoculation assay

Virus, cell line and organoids were treated as previously described (4). Briefly, the stock of SA11 rotavirus ($4.5*10^8$ $TCID_{50}$/ml) was used. MA104 cell line was inoculated with the diluted stock virus at MOI of 0.7 at 37℃ with 5 µg/mL of trypsin (Gibco, Paisley, UK) and 5% CO_2 for 15 min.

For inoculating organoids, collected organoids were incubated with trypsin pre-activated rotavirus at concentration of $4.5*10^5$ $TCID_{50}$/ml for 1.5 h followed by 4 times wash with PBS. Afterwards, for RNA and protein detection assay, organoids with no Matrigel remain were spun down at 500 g for 10 min to adhered to the bottom of 24-well or 48-well plate coated with Collagen R solution (SERVA, Heidelberg, Germany). Culture medium was added gently and organoids were incubated at 37℃ with 5% CO_2. Supernatant was harvested for secondary infection assay after 48 h incubation. Supernatant and organoids were harvested at different time points for RNA isolation

or Western blot assay. Infected organoids for observation or staining were mixed with Matrigel and seeded back to 24-well plate for continues culturing.

For secondary infection assay, Caco2 cells were washed, suspended in T75 flask and subsequently seeded into a 48-well plate (5×10^4 cells/well). Culture medium was discarded when cell confluence was approximately 80%, and cell monolayers were washed twice with PBS. 100 µL of serum-free DMEM medium, then pretreated supernatant were added and incubated at 37℃ with 5% CO_2 for 60 min for infection, followed by 4 times washing with PBS to remove un-attached viruses. Then, cells were incubated with maintenance medium with 1 µg/mL of trypsin at 37℃ with 5% CO_2.

$TCID_{50}$ assay

The titers of rotaviruses produced by organoids were determined by calculating the $log_{10}TCID_{50}$/mL in Ma104 cells using the method developing by Reed and Muench in 1938(6).

RNA isolation and sequencing, cDNA synthesis and qRT-PCR

Total RNA was isolated using Macherey-Nagel NucleoSpin® RNA II kit (Bioke, Leiden, Netherlands) and quantified using a Nanodrop ND-1000 (Wilmington, DE, USA). The quality of RNA was measured by Bioanalyzer RNA 6000 Picochip as quality-control step, followed by RNA sequencing performed by Novogene with paired-end 150 bp (PE 150) sequencing strategy. qRT-PCR assay were performed and glyceraldehyde 3-phosphate dehydrogenase (GAPDH) gene was used as housekeeping gene. Relative gene expression was normalized to GAPDH using the formula $2^{-\Delta\Delta CT}$ ($\Delta\Delta CT = \Delta CTsample - \Delta CTcontrol$). Template control and reverse transcriptase control were included in all qRT-PCR experiments. Primers for SA11 rotavirus are

TGGTTAAACGCAGGATCGGA as sense and AACCTTTCCGCGTCTGGTAG as antisense primer. The primers for the reference gene GAPDH are GTCTCCTCTGACTTCAACAGCG and ACCACCCTGTTGCTGTAGTAGCCAA as sense and antisense primer, respectively.

Determination of organoids cell death

The staining and scoring process was performed as previously(3). In short, minimum of 100 organoids were counted after 3 days of passaging. After 1.5 h incubation with SA11 rotavirus, organoids were cultured in Matrigel for 48 h followed by staining with propidium iodide (PI) (red, dead cells), Hoechst (blue, nuclear), and Calcein (green, live cells). Images were detected using EVOS FL cell imaging system (Thermo Fisher). Under fluorescence version, organoids in which red signal is more than green signal and also more than 50% of blue signal was counted as positive. On the contrary, organoids in which green signal is more than red signal and also more than 50% of blue signal was treated as negative or viable. Three random visions in each well have been chosen and organoids have been counted by total number and viable number. The proportion of deteriorated organoids was calculated as (viable/ total).

Western blot assay

Lysed cells were subjected to SDS-PAGE, and proteins were transferred to PVDF membrane (Immobilon-FL). SA11 rotavirus VP4 (1:1000, HS-2, mouse monoclonal; provided by professor Harry Greenberg, Stanford University School of Medicine, USA) was detected by western blot analysis and β-actin protein was detected as loading control (sc-47778, 1:1000, mouse monoclonal; Santa Cruz). The intensity of the immunoreactive bands of blotted protein was quantified by the Odyssey V3.0 software.

Immunofluorescence analysis

After rotavirus infection, organoids were harvested and fixed in 4% paraformaldehyde in PBS at 4℃ for 10 min. Fixed organoids were added into the CytoSpin II Cytocentrifuge (Shandon Scientifi Ltd, Runcorn, England), then spun down at 1000 rpm for 2 min. The slides containing organoids were rinsed 3 times with PBS for 5 min each, followed by treatment with 0.1% (vol/vol) Tritonx100 for 4 min. Subsequently, the slides were twice rinsed with PBS for 5 min, followed by incubation with milk-tween-glycine medium (0.05% tween, 0.5% skim milk and 0.15% glycine) to block background staining for 30 min. Slides were incubated in a humidity chamber with anti-rotavirus antibody (1:250, mouse monoclonal; Abcam) and anti-EpCAM antibody (1:250, rabbit polyclonal; Abcam) diluted in milk-tween-glycine medium at 4 ºC overnight. Slides were washed 3 times for 5 min each in PBS prior to 1 h incubation with 1:1000 dilutions of the anti-mouse IgG (H+L, Alexa Fluor® 594) and the anti-rabbit IgG (H+L, Alexa Fluor® 488) secondary antibodies. Nuclei were stained with DAPI (4, 6-diamidino-2-phenylindole; Invitrogen). Images were detected using Leica SP5 cell imaging system.

Neutralization assay

Neutralizing monoclonal antibody (MAb) HS-1 was gifted by professor Harry Greenberg, Stanford University School of Medicine, USA. Neutralization assay was performed as previous study(7). Briefly, rotavirus were activated by 5 µg/mL trypsin, followed by adding Mab in series of dilution (1:1000, 1:250, 1:100), then kept neutralizing for 2 h at 37 ºC and overnight at 4 ºC. After 48 h inoculation with organoids, RNA were isolated and detected by qRT-PCR.

Genome-wide transcriptomic analysis by RNA sequencing

Biliary organoids (n = 3) was infected or uninfected (as negative control) by rotavirus SA11. After 48 hours inoculation all samples were harvested and the total RNA was isolated using RNeasy Micro Kit (QIAGEN). The quantity of RNA was measured by a NanoDrop 2000. RNA sequencing was performed by Novogene with the paired-end 150-bp (PE 150) sequencing strategy. Gene expression was analyzed. The identification of differentially expressed genes is based on $P < 0.05$ and absolute values of $logFc > 1.5$. GSEA with the library of Wiki2019 was performed to reveal the alteration of signaling pathways. The complete dataset was deposited at Data Archiving and Networked Services (DANS). It is publically and freely available, but assess to the data may require account to login the database. (https://easy.dans.knaw.nl/ui/datasets/id/easy-dataset:179259; https://doi.org/10.17026/dans-27b-j8k9)

Statistics

The statistical significance of differences between means was assessed with the Mann-Whitney test(GraphPad Prism 5; GraphPad Software Inc., La Jolla, CA). The threshold for statistical significance was defined as $P \leq 0.05$.

References

1. **Cecílio AB, de Faria DB, de Carvalho Oliveira P, Caldas S, de Oliveira DA, Sobral MEG, Duarte MGR, de Souza Moreira CP, Silva CG, de Almeida VL.** 2012. Screening of Brazilian medicinal plants for antiviral activity against rotavirus. Journal of ethnopharmacology **141:**975-981.
2. **Knipping K, Garssen J, van't Land B.** 2012. An evaluation of the inhibitory effects against rotavirus infection of edible plant extracts. Virology journal **9:**137.
3. **Chen S, Ding S, Yin Y, Xu L, Li P, Peppelenbosch MP, Pan Q, Wang W.** 2019. Suppression of pyrimidine biosynthesis by targeting DHODH enzyme robustly inhibits rotavirus replication. Antiviral research **167:**35-44.
4. **Yin Y, Bijvelds M, Dang W, Xu L, van der Eijk AA, Knipping K, Tuysuz N, Dekkers JF, Wang Y, de Jonge J.** 2015. Modeling rotavirus infection and antiviral therapy using primary intestinal organoids. Antiviral research **123:**120-131.
5. **Huch M, Gehart H, van Boxtel R, Hamer K, Blokzijl F, Verstegen MMA, Ellis E, van Wenum M, Fuchs SA, de Ligt J.** 2015. Long-term culture of genome-stable bipotent stem cells from adult human liver. Cell **160:**299-312.
6. **Reed LJ, Muench H.** 1938. A simple method of estimating fifty per cent endpoints. American journal of epidemiology **27:**493-497.
7. **Ruggeri FM, Greenberg HB.** 1991. Antibodies to the trypsin cleavage peptide VP8 neutralize rotavirus by inhibiting binding of virions to target cells in culture. Journal of virology **65:**2211-2219.
